# Laminin Receptor-Avid Nanotherapeutic EGCg-AuNPs as a Potential Alternative Therapeutic Approach to Prevent Restenosis

**DOI:** 10.3390/ijms17030316

**Published:** 2016-03-01

**Authors:** Menka Khoobchandani, Kavita Katti, Adam Maxwell, William P. Fay, Kattesh V. Katti

**Affiliations:** 1 Department of Radiology, University of Missouri, Columbia, MO 65211, USA; khoobchandanim@health.missouri.edu (M.K.); KattiKK@health.missouri.edu (K.K.); 2 Institute of Green Nanotechnology, University of Missouri, Columbia, MO 65211, USA; 3 Department of Medicine, University of Missouri, Harry S. Truman Memorial Veterans Hospital, Columbia, MO 65211, USA; amn6f@health.missouri.edu; 4 Department of Medical Pharmacology and Physiology, University of Missouri, Columbia, MO 65211, USA; 5 Department of Physics, University of Missouri, Columbia, MO 65211, USA; 6 Department of Biological Engineering, University of Missouri, Columbia, MO 65211, USA; 7 University of Missouri Research Reactor, University of Missouri, Columbia, MO 65211, USA

**Keywords:** epigallocatechin-3-gallate, gold nanoparticles, green synthesis, atherosclerosis

## Abstract

In our efforts to develop new approaches to treat and prevent human vascular diseases, we report herein our results on the proliferation and migration of human smooth muscles cells (SMCs) and endothelial cells (ECs) using epigallocatechin-3-gallate conjugated gold nanoparticles (EGCg-AuNPs) as possible alternatives to drug coated stents. Detailed *in vitro* stability studies of EGCg-AuNPs in various biological fluids, affinity and selectivity towards SMCs and ECs have been investigated. The EGCg-AuNPs showed selective inhibitory efficacy toward the migration of SMCs. However, the endothelial cells remained unaffected under similar experimental conditions. The cellular internalization studies have indicated that EGCg-AuNPs internalize into the SMCs and ECs within short periods of time through laminin receptor mediated endocytosis mode. Favorable toxicity profiles and selective affinity toward SMCs and ECs suggest that EGCg-AuNPs may provide attractive alternatives to drug coated stents and therefore offer new therapeutic approaches in treating cardiovascular diseases.

## 1. Introduction

Nanotechnology will play a pivotal role in treating and preventing human diseases by improving drug delivery to target cells/organs and tissues [1,2,3,4,5,6,7]. Nanomaterials serve as effective carriers of diagnostic and therapeutic drug molecules to achieve optimum drug delivery for use in the treatment of cancer, cardiovascular diseases and various other human diseases and disorders [8,9,10,11,12,13]. Functionalized nanoparticles provide effective payload capacity for drug molecules, while displaying unique magnetic, electrical, and physical properties [14,15,16,17]. For example, surface chemistry of gold nanoparticles allows conjugation with receptor-specific antibodies, proteins, peptides and receptor-avid biomolecules and drugs to achieve target specificity and selectivity [18,19,20,21]. Among various types of nanoparticles, functionalized gold nanoparticles have assumed a major dimension in nanomedicine because of their biocompatibility and favorable surface chemistry [22,23,24,25,26]. A number of recent examples have demonstrated that functionalized gold nanoparticles have been used to achieve superior delivery pathways with consequent lowering/elimination of nonspecific uptake of drug molecules [27,28,29,30,31,32,33,34].

Atherosclerotic coronary artery disease (CAD) is the leading cause of death in the United States and globally [35,36]. Catheter-based delivery of balloon-expandable metal stents into coronary arteries narrowed by atherosclerotic plaque has become a mainstay of the treatment of patients with CAD. Stents provide a rigid scaffold that helps prevent re-narrowing of blood vessels (restenosis)-a problem that plagued balloon angioplasty in the pre-stent era [37]. However, despite remarkable technological advances, significant limitations remain in stent-based treatment of CAD [38,39,40,41]. For example, stents often cannot be deployed in small or tortuous arteries or at vessel branch points. In addition, drugs that are coated on stents to inhibit cell proliferation in the vascular wall (the process that drives restenosis) also inhibit the regrowth of endothelial cells, which line the inner surface of the vessel. Delayed endothelial healing renders the artery vulnerable to blood clot formation (thrombosis), which can abruptly obstruct blood flow and cause myocardial infarction [42]. Recent clinical trials in patients treated with drug-eluting stents (DES) confirm the failure of this cardiovascular treatment modality in a significant percentage of patients [43]. DES have been linked to severe risks of blood clots which could develop months or even years after the stent is implanted [44]. As there are over one million DES being implanted annually, experts have estimated that over 2000 people may die each year due to the side effects of DES [45]. Several clinical investigations have concluded that approximately one out of every 200 patients who receive a DES will experience a blood clot between six months and four years after the stent is implanted. Prior studies of late stent thrombosis show that these blood clots lead to a major heart attack or death approximately 70% of the time, with a fatality rate of 30% to 45% [43].

The development of in-stent restenosis (ISR) after DES implantation is a significant clinical problem. Therefore, the development of safe and effective alternatives to DES to deliver wound healing, anti-inflammatory and plaque stabilizing drugs to blood vessels are needed. We hypothesize that gold nanoparticles capable of selectively delivering drugs within narrow blood vessels will bring about a paradigm shift as alternatives to drug-coated stents. Toward this objective, we have discovered a number of functionalized nanoparticles including epigallocatechin-3-gallate (EGCg)-coated-gold nanoparticles (EGCg-AuNPs) in our laboratory [22,24,26,29,32,33,46,47]. The polyphenolic EGCg from tea extract has been shown to exert anti-thrombotic, anti-inflammatory and anti-oxidant activities [33,48,49,50]. EGCg conjugated gold nanoparticles are readily internalized by laminin receptor-positive cells, thus overcoming the hydrophobic barrier of cell membranes [33]. Laminin receptors are expressed on endothelial and smooth muscle cells that are found in abundance within the arterial walls [51]. Oxidative stress is implicated in the pathogenesis of neointimal hyperplasia and restenosis, and therefore effective delivery of powerful antioxidants, such as EGCg, could play significant roles to repair vascular injury aiding the prevention and treatment of neointimal hyperplasia and restenosis.

Therefore, we hypothesized that EGCg-AuNPs will show laminin receptor specificity and internalize through endocytosis into smooth muscle and endothelial cells, and thus will serve as an effective delivery vehicle of the therapeutic agent (EGCg), within vascular cells. We have validated our hypothesis through extensive experiments, which unequivocally provide experimental evidence for the internalization of EGCg-AuNPs within smooth muscle and endothelial cells. We herein report: (i) complete *in vitro* analysis for cellular internalization of EGCg-AuNPs by dark field microscopy and transmission electron microscopy (TEM); (ii) *in vitro* cytotoxic efficacy of free EGCg and the EGCg-AuNPs against human umbilical vein endothelial cells (HUVECs), human aortic endothelial cells (HAECs), and human coronary artery smooth muscles cells (HCASMCs); and (iii) effect of free EGCg and EGCg-AuNPs on endothelial and smooth muscles cells migration by scratch assay. The overall implications of EGCg-AuNPs as an effective strategy to prevent adverse vascular remodeling are presented.

## 2. Results

### 2.1. Synthesis and Characterization of Epigallocatechin-3-Gallate Conjugated Gold Nanoparticles (EGCg-AuNPs)

**Figure 1:**
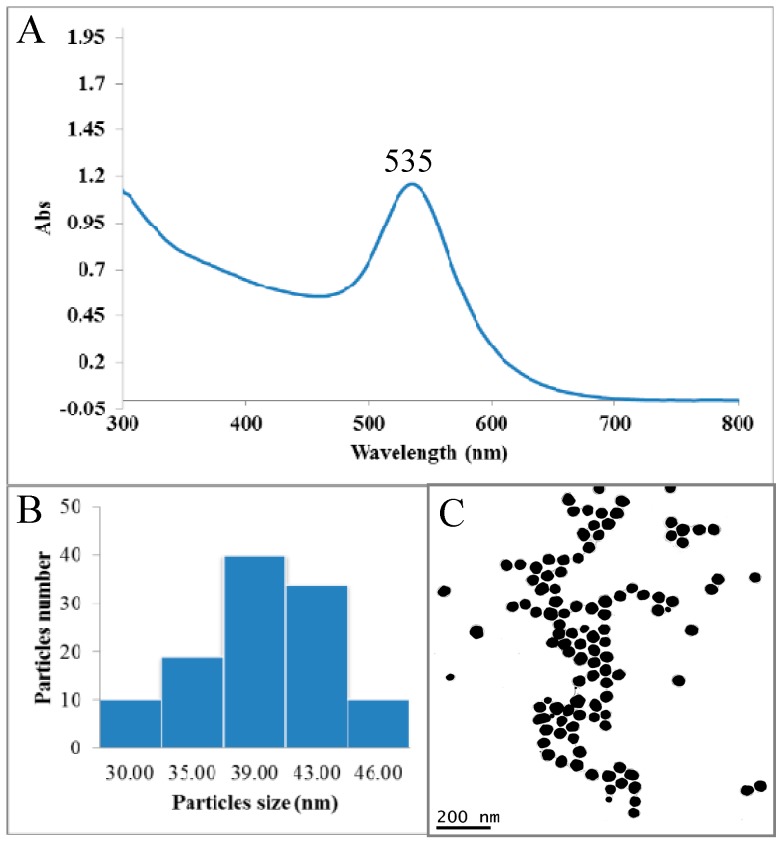
(**A**) UV–Vis absorption spectra of epigallocatechin-3-gallate conjugated gold nanoparticles (EGCg-AuNPs); (**B**) Size distribution histogram of gold nanoparticle solution; (**C**) Metallic core size of AuNPs by transmission electron microscopy.

Spectrophotometric analysis of EGCg-AuNPs revealed the characteristic surface plasmon resonance (SPR) band was observed at 535 nm, which confirmed the formation of gold nanoparticles (Figure 1). TEM analysis showed that EGCg-AuNPs are spherical, mono disperse, and homogeneous with mean diameter 40 ± 5 nm. The hydrodynamic size and ζ potential charges of EGCg-AuNPs are 65 ± 5 nm and −35.0 ± 2 mV respectively (Table 1). The hydrodynamic size of AuNPs is greater than the core size, thus inferring the coating of EGCg onto gold nanoparticles. The negative ζ potential value (−35.0 ± 2 mV) of AuNPs provides the necessary repulsive forces for the particles to remain stable in solution. The concentration of gold metal in gold nanoparticles, as determined by atomic absorption spectrometry (AAS); was found to be 0.52 mg/mg (52%) of dried AuNPs. These results corroborated with the data obtained by AAS analysis and therefore confirmed that EGCg-AuNPs have ~48% coating of EGCg, These findings fully validate our previous findings published by Katti *et al.* [33,52]. EGCg-AuNPs are stable in various biological fluids at physiological pH under *in vitro* conditions. It is noticeable that these AuNPs are stable in aqueous media for over one year, thus corroborating the high ζ potential which keeps these nanoparticles stable against agglomeration. It is very significant that EGCg serves a dual role of transforming the gold salt to the corresponding nanoparticles (through chemical reduction) and also as an effective encapsulant around the nanoparticles providing optimum *in vitro* and *in vivo* stability [33].

### 2.2. Cellular Internalization Study

**Figure 2:**
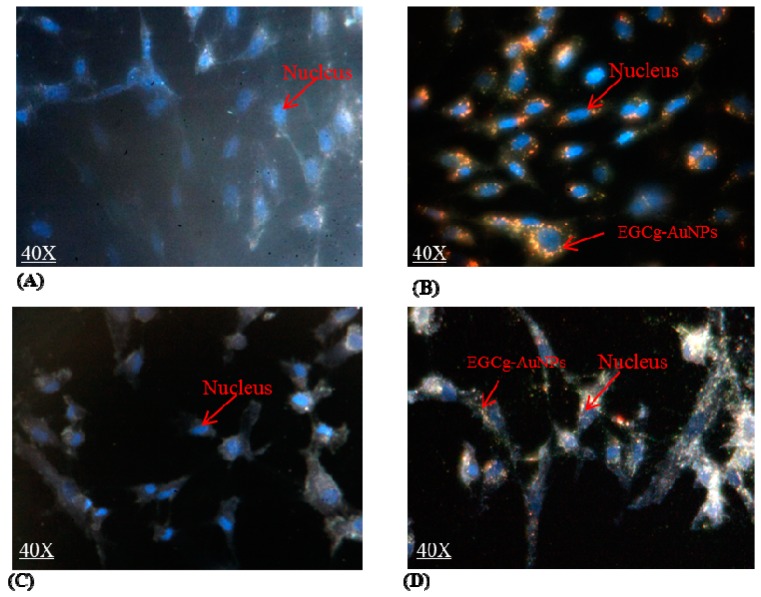
Cellular internalization of EGCg gold nanoparticles; images were captured via Cyto Viva dark field microscope. (**A**) human umbilical vein endothelial cells (HUVECs) untreated; (**B**) HUVECs treated with EGCg-AuNPs 20 μg/mL; 2 h; (**C**) human coronary artery smooth muscles cells (HCASMCs) untreated; (**D**) HCASMCs treated with EGCg-AuNPs 20 μg/mL; 2 h. HUVECs: Human umbilical vein endothelial cells, HCASMCs: Human coronary artery smooth muscle cells. Images were captured at 40× magnification.

**Figure 3:**
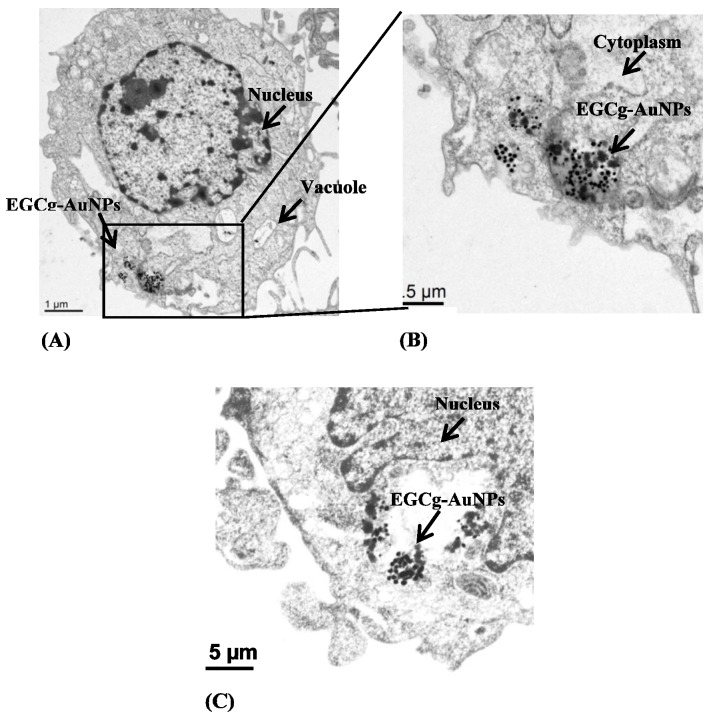
Cellular internalization of EGCg gold nanoparticles in HUVECs (**A**,**B**) and HCASMCs (**C**) with concentration 20 μg/mL, 2 h; images were captured by transmission electron microscopy. HUVECs (Human umbilical vein endothelial cells); HCASMCs (Human coronary artery smooth muscle cells).

The cellular internalization of EGCg-AuNPs in HUVECs and HCASMCs cells was evaluated using dark field optical microscopy and transmission electron microscopic techniques at various concentrations and time points. Dark field microscopic images unequivocally delineate that EGCg-AuNPs internalize effectively within HUVECs and HCASMCs within 2 h (Figure 2). TEM images unambiguously indicated that these nanoparticles are internalized into vacuoles and lysosomes of both the cell lines within 2 h (Figure 3). Our cellular internalization observations, in conjunction with the high level of expression of laminin receptors by HUVECs and HCASMCs cells [33,53], suggested that the high affinity and the endocytosis of EGCg-AuNPs is presumably mediated through laminin receptor expression by these cells (Figure 2 and Figure 3).

### 2.3. Laminin Receptor Blocking Studies

**Figure 4:**
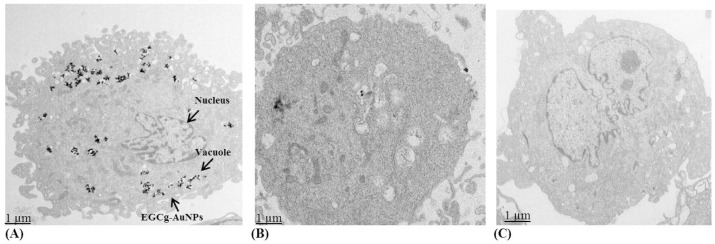
Cellular internalization of gold nanoparticles in HUVECs: (**A**) cells treated with GCg-AuNPs (50 µg/mL), 2 h; (**B**) cells pretreated with laminin receptor blocking antibody (ABLR), then treated with EGCg-AuNPs (50 µg/mL), 2 h; (**C**) cells treated with starch functionalized gold nanoparticles (50 µg/mL), 2 h; images were captured by transmission electron microscopy. HUVECs (human umbilical vein endothelial cells).

**Figure 5:**
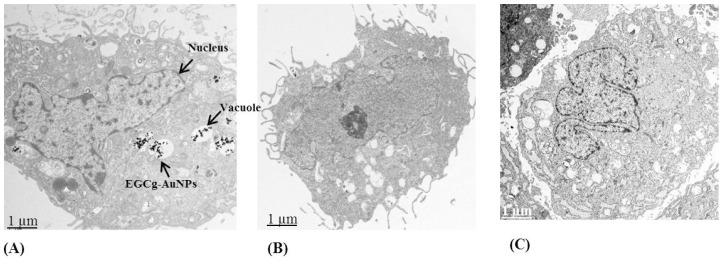
Cellular internalization of gold nanoparticles in HCASMCs : (**A**) cells treated with EGCg-AuNPs (50 µg/mL), 2 h; (**B**) cells pretreated with laminin receptor blocking antibody (ABLR), then treated with EGCg-AuNPs (50 µg/mL), 2 h; (**C**) cells treated with starch functionalized gold nanoparticles (50 µg/mL), 2 h; images were captured by transmission electron microscopy. HCASMCs (human coronary artery smooth muscle cells).

To demonstrate the laminin receptor affinity of EGCg-AuNPs towards ECs and SMCs, we performed extensive laminin receptor blocking studies using the laminin receptor specific antibody (ABLR). ABLR binds to laminin receptors to inhibit ligand binding. We incubated ECs and SMCs cells independently with ABLR for 60 min, followed by treatment with EGCg-AuNPs for 2 h. TEM analysis was performed after receptor blocking and subsequent incubation with EGCg-AuNPs did not reveal the presence of gold nanoparticles within these cells Figure 4B. In sharp contrast, significant endocytosis of EGCg-AuNPs was observed in the cells where laminin receptors were not blocked (Figure 4A). Likewise, laminin receptor blocking studies using the ABLR antibody on SMCs clearly demonstrated that with pre blocking of the laminin receptors, there was minimal/no uptake of EGCg-AuNPs (Figure 5B); whereas significant endocytosis of EGCg-AuNPs was observed in the cells where laminin receptors were not blocked (Figure 5A).

To further establish the laminin receptor specificity of EGCg-AuNPs, we compared endocytosis patterns with a nonspecific gold nanoparticulate construct. For example, starch functionalized gold nanoparticles are nonspecific and show no affinity toward laminin receptors. The TEM images shown in Figure 4C and Figure 5C clearly demonstrate that starch-conjugated gold nanoparticles are not internalized into ECs and SMCs, respectively. Therefore, these blocking studies provide compelling support for the selectivity and specificity of EGCg-AuNPs toward laminin receptors, which are expressed on ECs and SMCs.

### 2.4. In Vitro Cell Viability and Cytotoxicity Profile of EGCg and EGCg-Coated Gold Nanoparticles

**Figure 6:**
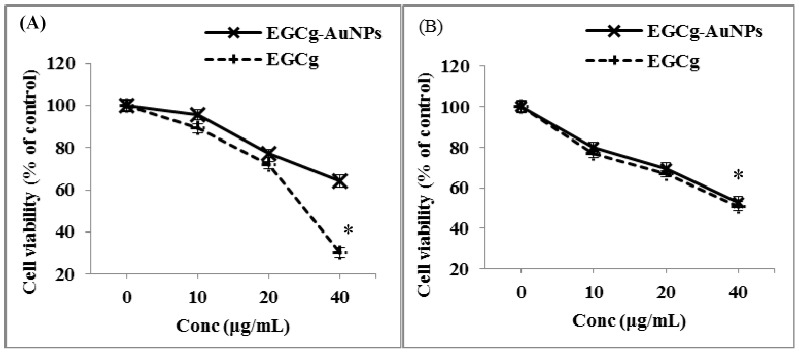
Effect of EGCg-AuNPs and free EGCg on endothelial and smooth muscles cells viability; (means ± SE; *n* = 3), cells treated for 24 h. (**A**) HUVECs (Human umbilical vein endothelial cells): * *p* < 0.05 *vs.* EGCg-AuNPs at same concentration (40 µg/mL); (**B**) HCASMCs (Human coronary artery smooth muscle cells): * *p* > 0.05 *vs.* EGCg-AuNPs at same concentration (40 µg/mL).

We studied the effects of free EGCg and EGCg-AuNPs on the viability of HUVECs and HCASMCs. Free EGCg inhibited the proliferation of endothelial cells by 70% ± 2.2% (Figure 4A). In contrast, EGCg-AuNPs showed 36% ± 2.8% inhibition of endothelial cells proliferation at concentrations of 40 μg/mL (Figure 6A). These results clearly reveal that the free EGCg showed significant toxic effects on endothelial cells as compared to the EGCg-AuNPs. The inhibitory effect of EGCg and the EGCg-AuNPs was further tested against the smooth muscles cell line. The results suggest that free EGCg and EGCg-AuNPs both inhibited the proliferation of smooth muscles cells at concentration of 40 μg/mL (Figure 6B). Our results indicate that EGCg and EGCg-AuNPs showed a statistically significant inhibitory effect on smooth muscles cells proliferation (~50% inhibition).

**Figure 7:**
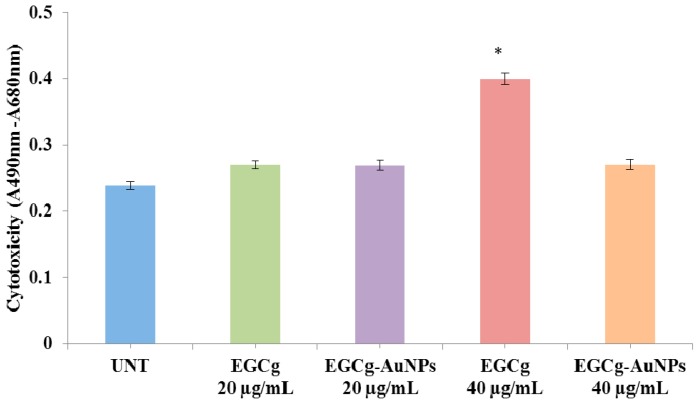
Cytotoxic effect of EGCg-AuNPs and free EGCg on human aortic endothelial cells (HAECs) with 24 h incubation time (mean ± SE; *n* = 3). * *p* < 0.05 *vs.* EGCg-AuNPs at same concentration.

We further investigated the possible cytotoxic effects of the free EGCg and the corresponding EGCg-conjugated gold nanoparticles on endothelial cells through LDH (lactate dehydrogenase) assay. The cytotoxicity was measured in terms of the amount of LDH released from the damaged cells, which is a sensitive marker for cellular toxicity. EGCg induced significant cytotoxic effect as compared to EGCg-AuNPs at concentration of 40 μg/mL (Figure 7).

### 2.5. Effect of EGCg and EGCg-AuNPs on Endothelial and Smooth Muscle Cells Migration

**Figure 8:**
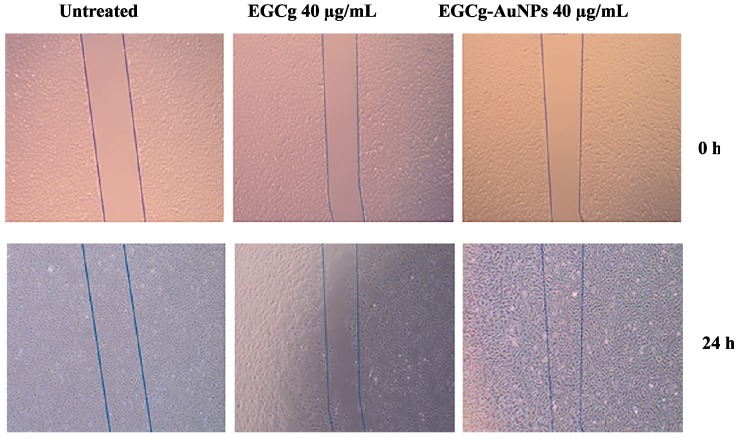
Inhibitory effects of EGCg-AuNPs and free EGCg on human aortic endothelial cells (HAECs) migration. Images were captured at 4× magnification.

**Figure 9:**
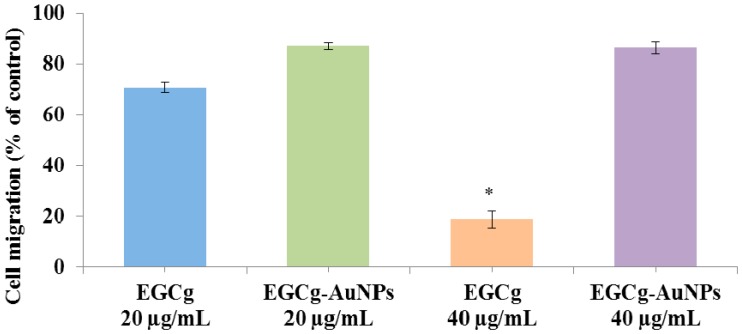
Effects of EGCg-AuNPs and free EGCg on HAECs migration with 24 h incubation time; mean ± SE (*n* = 3). * *p* < 0.05 *vs* EGCg-AuNPs at same concentration. HAECs (Human aortic endothelial cells).

We investigated whether EGCg and EGCg-conjugated gold nanoparticles affect endothelial cell migration. Cells were separately treated with free EGCg and EGCg-AuNPs at concentrations of 20 and 40 μg/mL. EGCg-AuNPs did not significantly inhibit the migration of HAECs, whereas free EGCg significantly inhibited the migration of HAECs by 81% ± 3.4% at the same concentration (Figure 8 and Figure 9). These data are statistically significant and comparable to the control group, where we observed that the scratched area was fully populated with cells within 24 h. These results corroborate that EGCg-AuNPs did not affect the migration of endothelial cells whereas the free EGCg inhibited the migration of endothelial cells at concentration of 40 μg/mL (Figure 9).

**Figure 10:**
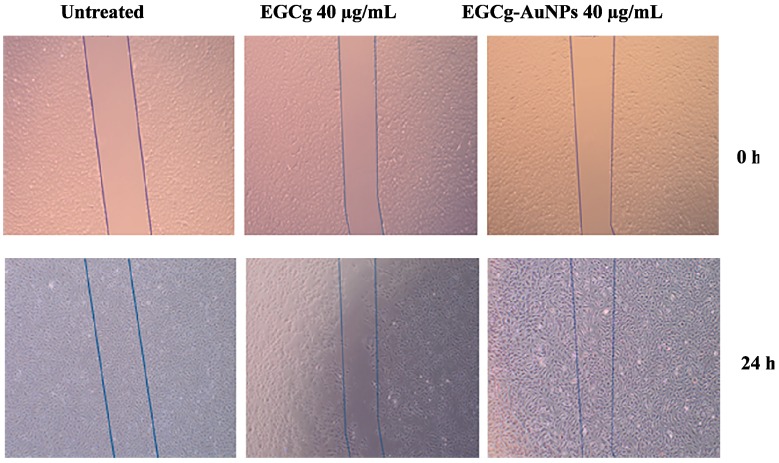
Inhibitory effects of EGCg-AuNPs and free EGCg on human coronary artery smooth muscles cell (HCASMCs) migration. Images were captured at 4× magnification.

**Figure 11:**
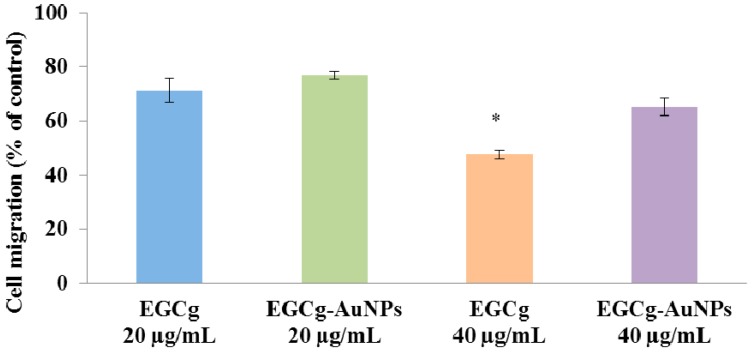
Effects of EGCg-AuNPs and free EGCg on HCASMCs migration with 24 h incubation time; mean ± SE (*n* = 3). * *p* < 0.05 *vs* EGCg-AuNPs at same concentration. HCASMCs (Human coronary artery smooth muscle cells).

We also studied the effects of EGCg-AuNPs and free EGCg on SMC migration at a concentration of 40 µg/mL, free EGCg inhibited the migration of SMCs by 52% ± 1.7% whereas EGCg-AuNPs inhibited the migration of SMCs by 35% ± 3.2% (Figure 10 and Figure 11). The results obtained for EGCg and EGCg-AuNPs were statistically significant and comparable to the effects observed with the control group, where we observed that the denuded area was populated with cells within 24 h (Figure 10).

## 3. Discussion

The nanotechnology revolution is making major impacts in biomedical sciences as numerous examples have demonstrated applications of engineered nanoparticles as effective delivery vehicles for drugs, and for theranostic agents [54,55]. While a plethora of tumor specific nanoparticles are being used in early diagnostics and therapy of cancer [56], the utility of functionalized nanoparticles as diagnostic or therapy agents in the treatment of cardiovascular diseases has remained relatively unexplored [57]. As part of our ongoing research on the intervention of nanotechnology in solving vexing medical problems, we are therefore, interested in exploring if biocompatible gold nanoparticles can be used in the treatment of cardiovascular diseases. In this context, we are focusing on the utility of gold nanoparticles functionalized with epigallocatechin gallate (EGCg), obtained from a naturally occurring polyphenol from tea, as potential nano cardiotherapeutic agents [33]. EGCg-AuNPs were produced, through a novel green nanotechnology process discovered by Katti *et al.*, [33] by simple mixing of gold salt with EGCg solution at room temperature in aqueous media. These nanoparticles have been fully characterized with hydrodynamic sizes of 65 ± 5 nm which is the optimum size for cell penetration for use in various biomedical applications [27]. The negative ζ potential value (‒35.0 ± 2 mV) for EGCg-AuNPs indicates that these nanoparticles are highly stable due to the repulsive forces exerted by multiple groups of –OH functionalities from the polyphenols, which are conjugated to the AuNPs. The repulsive force from the –OH groups keeps the EGCg-AuNPs repelling each other strongly and thus prevents agglomeration of these nanoparticles. Our extensive *in vitro* investigations have demonstrated that these nanoparticles are stable in various biological media e.g., human serum albumin and in different aqueous dilutions that mimic cellular concentrations found in biological profiles.

In order to understand the possible interaction between EGCg-conjugated gold nanoparticles and endothelial/smooth muscles cells, we have performed cellular internalization studies using dark field microscopy and TEM techniques. This study was performed to understand whether these nanoparticles are involved in the phagocytosis and/or receptor mediated endocytosis pathways. Our results revealed that EGCg-AuNPs internalize into both SMCs and ECs within 2 h. Furthermore, the specificity of EGCg-AuNPs for laminin receptors expressed on ECs and SMCs was confirmed by receptor blocking studies. The TEM analysis clearly showed reduction of EGCg-AuNPs uptake in ECs and SMCs cells after blocking the laminin receptors. The results were comparable with the samples treated with EGCg-AuNPs, without blocking LR, where the uptake of AuNPs was significantly higher. We have further investigated the receptor specificity of EGCg-AuNPs by comparing the endocytosis pattern with non-specific gold nanoparticles. The TEM images indicated that there was no uptake of AuNPs in ECs and SMCs. These results together corroborate laminin receptor-mediated endocytosis of EGCg-AuNPs because it is well established that both SMCs and ECs express this receptor [33,53].

In cardiovascular diseases, endothelial and smooth muscles cells play key roles in the pathogenesis of arteriosclerosis and anti-angiogenesis therapy. The proliferation and migration of smooth muscles cells are of critical significance leading to neointima formation after vascular injury [58]. Neointima formation is mediated by thrombotic and inflammatory mediators, growth factors, cytokines and oxidative stress [59]. In this investigation, we have tested the effects of EGCg conjugated gold nanoparticles against endothelial and smooth muscles cells in terms of viability, toxicity, and migration using *in vitro* systems. The cytotoxicity data suggests that EGCg-AuNPs at 40 μg/mL concentration did not affect endothelial cells proliferation whereas free EGCg was toxic and inhibited the proliferation of endothelial cells. The cytotoxic effect was also confirmed by the LDH assay against the endothelial cells. The data obtained from LDH assay supported that the EGCg-AuNPs did not show any toxicity towards endothelial cells whereas the free EGCg showed significant toxicity. It is important to note that the results obtained from EGCg-AuNPs were comparable with the control group, where endothelial cells were viable and healthy.

Our studies of *in vitro* migration revealed selectivity of the toxic effects of EGCg and the nontoxic nature of the corresponding EGCg-AuNPs on human aortic endothelial cells (HAECs). The EGCg-AuNPs treated HAECs showed a migration pattern that was similar to the control group at concentrations of 40 μg/mL at 24 h. Under similar experimental conditions, we observed reproducible inhibition in the migration of human aortic endothelial cells by 81.4% when the free EGCg was used. These results provide compelling evidence that EGCg-AuNPs are non-toxic towards endothelial cells as compared to the free EGCg. These results, taken together, clearly demonstrate that the coating of EGCg phytochemical on gold nanoparticles results in their effective delivery into the cellular matrix with no toxicity towards endothelial cells as compared to the free EGCg.

The abundance of smooth muscle cells within the arterial walls and also their vulnerability for damage during artery ruptures prompted us to investigate the inhibitory effects of EGCg-AuNPs and their comparisons with free EGCg against these cells. The proliferation and migration of smooth muscles cells lead to neointimal hyperplasia resulting in the thickening of the arterial wall. Therefore, it is important to inhibit the over growth of SMCs without damaging the ECs. From our experiments, we have confirmed the effect of EGCg-AuNPs and the free EGCg on SMCs viability and migration. The cell viability data revealed that both the free EGCg and EGCg-AuNPs significantly inhibit the proliferation of smooth muscles cells at 40 μg/mL concentration. This infers that the free EGCg and EGCg-AuNPs significantly delay the migration of smooth muscles cells as revealed by the minimum population of cells in the denuded area (Figure 10). In contrast, the control group of SMCs, which were not treated either with EGCg or EGCg-AuNPs, under similar experimental conditions showed considerable population of cells within the denuded area (Figure 10). These results, taken together, unequivocally confirm that both EGCg-AuNPs and free EGCg inhibit the migration of smooth muscles cells. These results suggest that EGCg-conjugated gold nanoparticles may have the potential for use as alternatives to drug-eluting stents in the treatment of cardiovascular diseases.

The selective efficacy of EGCg towards ECs and SMCs could be attributed to its molecular structure, which includes a plethora of hydroxyl groups and galloyl group [60]. Effects of EGCg for their cardioprotective properties as well as for their efficacy as anti-atherosclerotic and anti-hypercholesterolemic characteristics have been explored in the past [61,62]. Our previous investigations related to the *in vitro* and vivo antitumor efficacy have demonstrated that EGCg conjugated onto AuNPs has better efficacy compared to the free EGCg due to the larger surface area of AuNPs [22,33]. A number of additional studies have also reported that EGCg and related polyphenols present in green tea affects SMCs proliferation via activating p53, by down regulating NF-κB, or by suppressing mitogen activated protein kinase pathway [63,64]. A study reported in the literature depicts that EGCg decreases oxidative stress and inflammation in ECs by reducing NADPH oxidase expression at the transcriptional level [65]. Our findings corroborate earlier observations reported by Han and co-workers [66], related to the effects of EGCg on ECs and SMCs migrations. Our present study has confirmed the laminin receptor affinity, which makes these gold nanoparticles selective towards ECs and SMCs. This is an important finding corroborating significant selectivity of EGCg-functionalized gold nanoparticles. The targeting ability of EGCg toward laminin receptors, which are over expressed in SMCs, is presumably responsible for the selective and effective delivery of EGCg through AuNPs.

## 4. Experimental Section

### 4.1. Materials

Epigallocatechin gallate (EGCg), gold salt (NaAuCl_4_), dyes-Trypan blue, and DAPI (4’,6-diamidino-2-phenylindole) were obtained from Sigma, St. Louis, MO, USA. Fetal calf serum and TryplE, Media 200 and Media 231, low serum growth supplement (LSGS), and smooth muscle growth supplement (SMGS) were obtained from Life Invitrogen, New York city, NY, USA. Human umbilical vein endothelial cells (HUVECs), human aortic endothelial cells (HAECs), and human coronary artery smooth muscles cells (HCASMCs) were obtained from American Type Culture Collection (ATCC; Manassas, VA, USA). The protocol for the “in vitro cell culture experiments” was approved by MU Institutional Biosafety Committee (protocol #08-04), University of Missouri, Columbia, MO, USA. 

### 4.2. Synthesis of EGCg Coated Gold Nanoparticles (EGCg-AuNPs)

The EGCg-AuNPs were synthesized using a slightly modified procedure. [33] Briefly, EGCg (2.2 mg) was added to 6 mL of DI water in a scintillation vial and stirred for 20 min at room temperature (rt) on a magnetic stirrer. Then, 100 μL of 0.1 M NaAuCl_4_ solution was added and the color of the solution turned to ruby-red within 5 min, indicating the formation of gold nanoparticles. Nanoparticles were centrifuged twice at 8000 rpm at 12 °C for 15 min to remove the unreacted EGCg. The gold nanoparticles were characterized and were stored at 4 °C. The treatment concentrations of EGCg-AuNPs were calculated on the basis of amount of EGCg within the nanoparticles. However, for the free EGCg, the concentrations were the same as the EGCg concentration present in the EGCg-AuNPs. The amount of gold in the nanoparticles was calculated by atomic absorption spectrometry technique (AAS, Perkin Elmer, MA, USA). The amount of EGCg in the AuNPs was determined by AAS and was also quantitatively determined through chemical methods as reported by Katti *et al.* [33,52].

### 4.3. Characterization of EGCg-AuNPs

Transmission Electron Microscopic (TEM) images were obtained on a JEOL 1400 TEM (JEOL, LTE, Tokyo, Japan). The absorption measurements were made using a Varian Cary 50 UV-Vis spectrophotometer, Shimadzu, Columbia, MD, USA. The hydrodynamic diameter and ζ potentials were measured using Zetasizer Nano S90 (Malvern Instruments Ltd., Westborough, MA, USA).

### 4.4. Quantification of AuNPs by Furnace Atomic Absorption Spectroscopy (fAAS)

Gold metal content in EGCg-AuNPs was estimated by fAAS using a standard curve spanning 0–100 μg/mL. EGCg-AuNPs samples were digested with HNO_3_ and HCl (1:2 ratios) and kept into an oven at 85 °C overnight. After cooling at 25 °C, the digest was diluted in 1:10 ratio with ultrapure water for analysis. Quality-control materials (duplicates, spikes, and instrument-calibration verification) were within appropriate ranges.

### 4.5. Endocytosis and Cellular Uptake Assay of EGCg-AuNPs by Dark Field Microscopy

The *in vitro* cellular internalization (endocytosis) analysis of EGCg-AuNPs was performed by dark field cytoviva microscopic technique. Ultra clean and sterile cover slips were kept in 6 well plates. The HUVECs and HCASMCs (5 × 10^5^ cells) were seeded into 6 well plates in Media 200/231 separately and incubated for 24 h in CO_2_ incubator at 37 °C. EGCg-AuNPs (10 and 20 μg/mL) were added to cells followed by 2 h of incubation at 37 °C. The cells were washed 10–12 times with 1× PBS, and fixed with 4% para-formaldehyde (PFA). Cells were further washed 2 times with cold 1× PBS. Slides were prepared by using DAPI nuclear dye and observed under CytoViva dark field microscope coupled with dual mode fluorescence. Cell morphology was initially observed, followed by uptake of nanoparticles. Images were captured via Dage Imaging Software, (CytoViva Inc., Auburn, AL, USA) at 40× magnification.

### 4.6. Cellular Internalization of EGCg-AuNPs by Transmission Electron Microscopic (TEM)

The HUVECs & HCASMCs cells (5 × 10^5^ cells) were seeded into 6 well plates in Media 200/231 separately and allowed to adhere for 24 h in CO_2_ incubator at 37 °C. The media was replaced with EGCg-AuNPs (10 and 20 μg/mL) containing medium and incubated for 2 h at 37 °C. The cells were washed 12 times with PBS, centrifuged into small pellets, and fixed with 2% glutaraldehyde and 2% paraformaldehyde in sodium cacodylate buffer (0.1 M) and stored at 4 °C for further use. The cells were further fixed with 1% buffered osmium tetraoxide in 2-Mercaptoethanol buffer and dehydrated in graded acetone series and embedded in Epon-Spurr epoxy resin. Sections were cut at 85 nm using a diamond knife (Diatome, Hatfield, PA, USA). The sections were stained with Sato’s triple lead stain and 5% aqueous uranyl acetate for organelle visualization. The cellular samples were examined, for endocytosis of EGCg-AuNPs, on JEOL 1400 TEM microscope (JEOL, Peabody, MA, USA) operated at 80 kV at the Electron Microscopy Core Facility, University of Missouri (Columbia, MO, USA).

We have also evaluated the receptor binding affinity of EGCg-AuNPs towards ECs and SMCs by blocking laminin receptors. ABLR (Laminin receptor blocking antibody) is well known to bind to laminin receptors. ECs and SMCs were incubated independently with ABLR (10 µg/mL) for 60 min in order to block the laminin receptors of these cells followed by the treatment with EGCg-AuNPs for 2 h. The samples were processed and images were captured on JEOL 1400 TEM microscope (JEOL).

### 4.7. Cell Viability Assay

The *in vitro* cytotoxicity evaluation of EGCg-AuNPs and free EGCg was determined using MTT kit (Promega). The intensity of developed color was measured by micro plate reader (BioTek, Winooski, VT, USA) operating at 570 nm wavelength. Percent cell viability was calculated by using the formula: (T/C) × 100, where C = Absorbance of control, T = Absorbance of treatment.

### 4.8. Cytotoxicity Assay

The lactate dehydrogenase leakage assay (LDH) was determined using a LDH Kit (Thermo Scientific, Waltham, MA, USA). The LDH activity and the absorbance were measured at 490 and 680 nm by ELISA reader (BioTek).

### 4.9. Cell Migration Assay

HAECs and HCASMCs (5 × 10^5^) suspended in serum containing Media 200/media 231 were added to 12-well tissue culture plates and incubated at 37 °C until the cells reached 95% confluence. Media was changed to serum starvation conditions using 2% serum containing media overnight. A linear “scratch” was made across the entire diameter of each well with a disposable pipet tip, which completely removed cells from a linear region of the well. Medium was changed with different dilutions of EGCg and EGCg-AuNPs and incubated for 24 h. Digital photographs of two segments of each scratch were taken at 4× magnification with a microscope, both immediately after creating the scratch and after 24 h. Images were imported into cellSens digital imaging software, (Olympus, Center Valley, PA, USA) which precisely recorded the *X*–*Y* axis coordinates of each image to ensure that identical regions of the well were being compared at 0 and 24 h. The number of cells that migrated into the scratch zone was counted and the average of the 3 regions was determined for each well.

#### Statistical Analysis

All experimental data were given as mean ± SE. Statistical analysis was carried out using the Student’s *t* test using Graph Pad Prism software online. A *p* value less than 0.05 was determined as statistically significant.

## 5. Conclusions

The experimental results of our investigation corroborate laminin receptor-avidity and effective internalization of EGCg-AuNPs into endothelial and smooth muscles cells, which possess a high abundance of laminin receptors. Our studies have further inferred that cellular internalization of EGCg-AuNPs drastically affected the growth and proliferation of plaque-causing smooth muscles cells with relative nontoxic effects towards endothelial cells. Therefore, antioxidant and anti-inflammatory effects of EGCg-AuNPs can be transported across endothelial cells for possible wound healing of damaged and ruptured arteries. Overall, our aforementioned studies and experimental results have allowed us to validate our hypothesis that EGCg-AuNPs could serve as promising nanoparticulate drug-coated alternatives to stents for use in the treatment of various cardiovascular diseases.

## Figures and Tables

**Table 1:** Physicochemical parameters of epigallocatechin-3-gallate (EGCg)-coated-gold nanoparticles (EGCg-AuNPs).

UV Visible Spectrophotometry	DLS	ζ Potential	TEM	AAS
535 nm	65 ± 5 nm	−35.0 ± 2 mV	40 ± 5 nm	0.52 mg Au/mg of AuNPs

DLS: Dynamic light scattering, TEM: Transmission electron microscopy, AAS: atomic absorption spectroscopy.
